# Epidemiology of sarcoptic mange in free-ranging vicuñas (*Vicugna vicugna*): a cross-sectional study in Andean highland communities in Peru

**DOI:** 10.1590/S1984-29612024030

**Published:** 2024-07-08

**Authors:** Marcos Enrique Serrano-Martínez, Gabriel Bazán Alcántara, Marco Enciso, Fahrid Huanca Mori, Luis Llanco Albornoz, Stef de Haan, Henry Juárez, Sthefany Aguilar Tejeda, Cristofer Cruz Camero, Cesar Burga-Cisterna

**Affiliations:** 1 Facultad de Medicina Veterinaria y Zootecnia, Universidad Peruana Cayetano Heredia, Lima, Peru; 2 Servicio Nacional Forestal y de Fauna Silvestre – SERFOR, Lima, Peru; 3 Ministerio de Desarrollo Agrario y Riego – MIDAGRI, Lima, Peru; 4 Facultad de Medicina Veterinaria, Universidad Nacional Mayor de San Marcos, Lima, Peru; 5 Escuela Profesional de Medicina Humana, Universidad Privada San Juan Bautista – UPSJB, Chincha, Peru; 6 Centro Internacional de la Papa – CIP, Lima, Peru

**Keywords:** Scabies, Sarcoptes, vicuña, South American camelids, peasant communities, Escabiose, Sarcoptes, vicunha, Camélidos sul-americanos, comunidades camponesas

## Abstract

Sarcoptic mange or scabies is a contagious parasitic skin disease that affects a wide range of domestic and wildlife species. A cross-sectional study was carried out to determine the prevalence, molecular identification, and characteristics of sarcoptic mange in vicuñas. A total of 3,274 vicuñas were examined. Following ritual harvesting events (“chaccus”) in 13 Andean communities. The presence of mange mites was determined by the skin scraping technique and confirmed by PCR analysis using specific primers for the ITS2 gene of *Sarcoptes scabiei*. The presence of mange mites was also confirmed by microscopy using samples taken from wallows. A data collection form was used to register the characteristics of the vicuñas sampled. The prevalence of sarcoptic mange was 4.9% (95% CI: 4.1 - 5.6%). All samples from wallows tested positive (9/9). Importantly, the presence of the species *S. scabiei* was molecularly confirmed. Adult females with regular body condition were more susceptible to sarcoptic mange, although the lesions were mild. This study confirms the presence of *S. scabiei* in semi-captive vicuñas and points to the possible role of wallows in the dissemination of *Sarcoptes* mites.

## Resumo

A sarna sarcóptica é uma doença parasitária contagiosa da pele que afeta uma ampla gama de espécies domésticas e selvagens. Um estudo transversal foi realizado para determinar a prevalência, identificação molecular e características da sarna sarcóptica em vicunhas. Foram avaliadas 3.274 vicunhas, capturadas durante o "Chaccus", em 13 comunidades rurais alto andinas do Peru. A presença do ácaro da sarna foi determinada pelo raspado de pele e confirmada pela técnica de PCR, com iniciadores específicos para o gene ITS2 de *Sarcoptes scabiei* e por microscopia nos chafurdeiros. Foi preenchida uma ficha de coleta de dados com as características das vicunhas analisadas. A prevalência de sarna sarcóptica em vicunhas foi de 4,9% (95% IC: 4,1 - 5,6%). Todas as amostras de chafurda testadas (9/9) foram positivas, e a presença de *S. scabiei* foi confirmada molecularmente. Fêmeas adultas com condições corporais regulares foram mais suscetíveis à sarna sarcóptica, embora as lesões fossem leves. O estudo confirma a presença de *S. scabiei* em vicunhas em semicativeiro de comunidades camponesas alto andinas do Peru que sofrem de sarna. Destaca-se, ainda, o papel das chafurdas das vicunhas na disseminação da sarna.

## Introduction

The vicuña (*Vicugna vicugna*) is a South American camelid, native to the Andes. Its management as a wild resource providing fiber is part of the development history of Peru and its neighboring countries. The communal custodianship of vicuña populations and their exploitation rights are exclusive to Peru’s highland communities (Lichtenstein et al., 2002). This wild camelid species has a high economic and social value for its fiber, which can reach a value of 1,500 US dollars per kilogram (Pacheco et al., 2019).

The vicuña has historically been affected by poaching and the introduction of domestic animals into its high-altitude habitat. The latter has contributed to environmental degradation, particularly overgrazing, and the spread of infectious and parasitic diseases. Amongst them, ectoparasitoses such as Acariasis (mange) have become increasingly important (Acebes et al., 2022). In domesticated South American camelids, a range of mite species have been reported as the agent of Acariasis, *Sarcoptes scabiei*, *Chorioptes* sp., and *Demodex* sp. for lamas (Curtis et al., 2001; Eo et al., 2010) and *Eutrombicula* sp. for alpacas in Peru (Gómez-Puerta et al., 2012).

Mange mites cause intense itching, inflammation, formation of scabs with dandruff, weight loss, and eventually death in animals (Astorga et al., 2018; Gómez-Puerta et al., 2013). Mange is a severe problem in camelids, caprines, wild felines, and canids in Europe, North America, and Asia (Astorga et al., 2018). In South America it affects alpacas, llamas, and vicuñas (Acebes et al., 2022). In Argentina, the occurrence of infestations in vicuñas with *S. scabiei* and *Psoroptes* sp. has been reported. The lesions have been characterized as having “parakeratotic" and "alopecic" forms with the type I (immediate) and type IV (delayed) hypersensitive reactions dominating (Ferreyra et al., 2022). In the San Guillermo National Park (San Juan, Argentina) it has been observed that mange can affect vicuñas and guanacos, reaching a prevalence of up to of 56% (Ferreyra et al., 2022), resulting in high rates of mortality (Ferreyra et al., 2022). In Peru, mange can reach prevalence of up to 26% (Bujaico, 2018) and generate economic losses of up to 80,000 soles (= US$ 21,917) in some highland communities (El Comercio, 2015). Yet, Gomez-puerta have highlighted the existence of a research gap and lack of data (Gómez-Puerta et al., 2013). Currently, the disease is considered the main cause of mortality in vicuñas, surpassing poaching (Halsby et al., 2017; Peru, 2014).

The treatment of mange is based on the use of injectable and topical drugs that include organophosphates, pyrethroids, and avermectins, among which ivermectin stands out. However, their constant use has generated resistance, as well as being potentially toxic and affecting the environment (Coles & Dryden, 2014). Ivermectin, the first-line drug against this type of infestation, has been evaluated as a treatment in alpacas and llamas in long-term schemes (D'Alterio et al., 2005; Castilla-Castaño et al., 2021). In recent years some cases of reduced efficiency (less than 86%) have been reported, even when used in combination with other topical treatments (Lusat et al., 2009; Astorga et al., 2018; Pollock et al., 2017). This situation is partly due to the inadequate use of this drug and in particular a lack of appropriate dosages as recommended for sheep, cattle, or pigs. The appropriate dosage required for domesticated and wild Andean camelids has been poorly investigated (Rowe et al., 2019). Studies on the pharmacodynamics of different drugs (avermectins) against mange in alpacas have shown the need for a higher dose compared to cattle in order to get similar therapeutic effects (Pollock et al., 2017).

The diagnosis of mange by microscopy provides valuable information for its control; however, molecular techniques are currently in use to identify the various species of mites that cause mange and understand the epidemiology of this skin disease (Nashat et al., 2018; Sastre et al., 2013). Thus, several of the mange-generating mite species, which appear to be different when analyzed by taxonomic keys, are similar in phylogenetic tests, suggesting that these parasites do not have specific hosts (Bochkov et al., 2014). Many studies on the taxonomic classification of mites based on genetic markers such as cytochrome (cox1 mtDNA), 28S rDNA, and ITS2, have proven quite useful for the phylogenetic grouping of these parasites and are necessary to confirm interspecies transmission (Wang et al., 2012; Zhao et al., 2020).

Regarding factors triggering mange in alpacas and llamas, some authors have reported that the severity of the lesions depends on environmental and nutritional factors (Castilla-Castaño et al; 2021). For example, zinc and copper deficiency can lead to more intense inflammatory reaction. Importantly, in the case of vicuñas, the limited literature and the infrequent handling of animals reduce the possibility to mitigate the risk factors of the disease and produce adequate tools for its effective control. The present study aims to investigate the occurrence of mange mites in vicuñas from typical highland communities using microscopy and molecular techniques, thereby contributing to our knowledge of the causal agents and severity of the disease in Peru.

## Materials and Methods

### Population and sample

**Figure 1:**
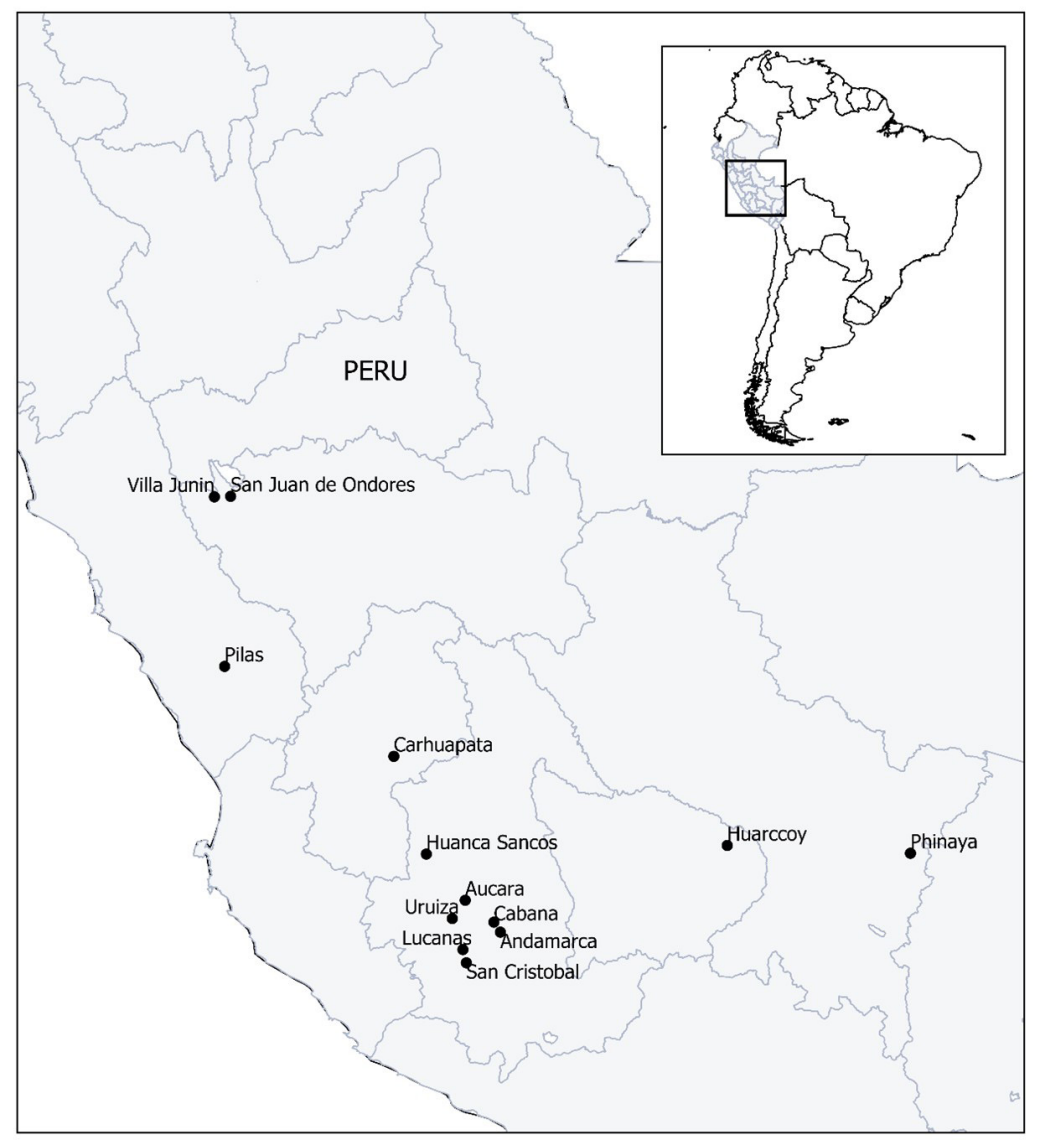
Map of Peru and geographic location of the Andean highland communities included in this study.

**Figure 2:**
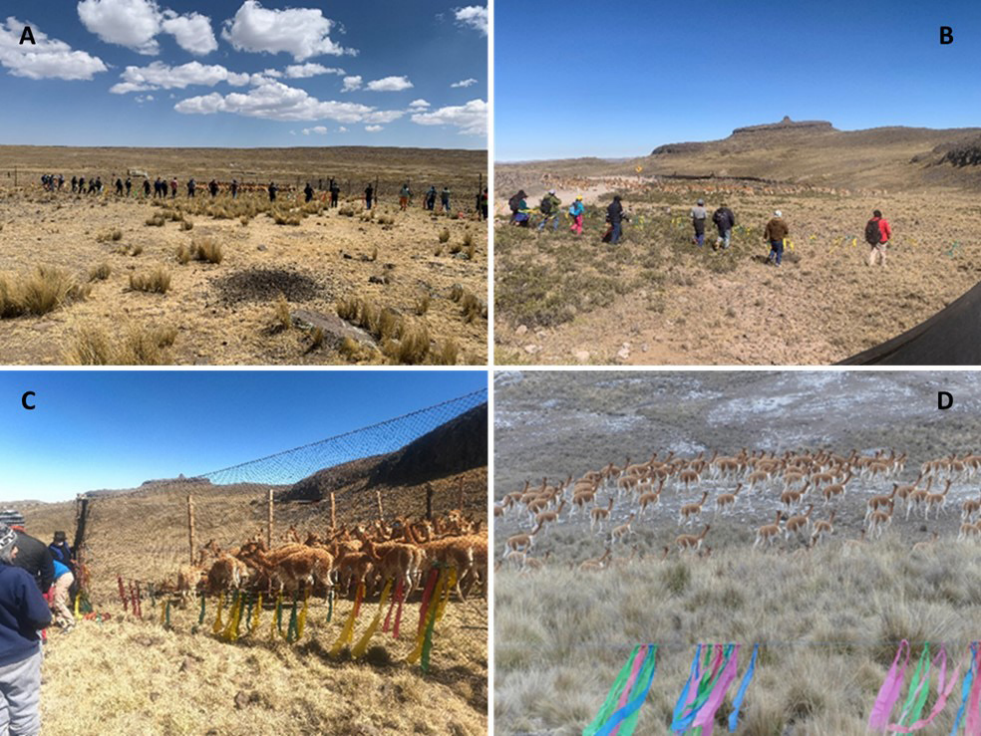
Vicuña´s Chaccu: (A-D): sighting, fence, capture, and release of vicuñas.

A total of 3,274 vicuñas were captured and individually handled. The investigated groups originated from 13 highland communities in the departments of Apurímac (Huarccoy), Ayacucho (Andamarca, Aucara, Cabana, Huancasancos, Lucanas, San Cristóbal, and Uruiza), Cusco (Phinaya), Lima (Santiago de Pilas), Junin (Villa de Junin and Ondores) and Huancavelica (Carhuapata) (Figure 1). To reduce the handling stress of the vicuñas, all animals included in this study were rounded up during the annual capture and shearing ritual called "chaccu" (Figure 2), and clinically examined under veterinary supervision. The sampling was carried out from July to September 2021. During this period the average environmental temperature was 24.2 °C and the relative humidity of 81.5% (Senamhi, 2021).

### Identification of mites in skin scrapings

Skin scrapings were collected from different alopecic areas of the vicuñas with mineral oil, and subsequently stored in sterile wide-mouthed flasks with a register of the animal's data, origin, and date of the specific sample. Sampled were refrigerated during their transport to the Animal Parasitology Laboratory of the Cayetano Heredia Peruvian University in Lima. When the samples arrived at the laboratory, they were treated with 10% KOH, placed on slides, and covered with coverslips to be observed under a microscope at 10X and 40X.

### Identification of mites in wallows

Soil samples from wallows were collected from three out of the thirteen highland communities included in the study (Huarccoy, Andamarca, and San Cristóbal). Samples of 4 kg of soil for each wallow were placed in sealed bags and transported without refrigeration to the laboratory in Lima. Soil samples were processed by sedimentation techniques (Yahia et al., 2023). Subsequently, mites were placed on slides, and covered with coverslips to be observed under a microscope at 10X and 40X. Mites obtained from the sediment were identified using standard microscopic techniques, Additionally, the parasite load was evaluated by quantifying the average number of mites present in three 100 g aliquots of each wallow soil sample.

### Molecular characterization of mites

Samples positive for scabies mites identified by microscopy were evaluated by polymerase chain reaction (PCR) to confirm the parasitic species. The oligonucleotide primers targeted the ITS - 2 region of *S. scabiei*: Forward: TGTTAGTAGTAGCTCTATGAGAA; Reverse: TCGCTTGATCTGAGGTCG) (Fraser et al., 2018). The DNA was extracted with the Wizard® SV Genomic DNA Purification System kit, following the manufacturer's instructions. The DNA obtained was quantified with the Thermo Scientific™ NanoDrop™ Lite Spectrophotometer (Thermo Fisher Scientific, USA) and frozen at -20 °C until processing. To determine the mite species, the ITS-2 region was amplified following the next PCR protocol: in a final volume of 25 uL, 12.5 uL of GoTaq Master mix (Invitrogen) (containing 0.5 U of Taq polymerase, 2 mM of MgCl2 and 25 nMol dNTPs), 2 uL of blank DNA, 1.25 uL of each primer, and 3 uL of ultrapure water (MiliQ). The amplification conditions were 95°C for 2 min (initial denaturation), then 35 cycles each with an initial 30 sec at 95°C (denaturation), 30 sec at 60°C (annealing), 72°C for 1 min (extension) and a final extension of 72°C for 10 min. The amplified samples were analyzed by electrophoresis in 2% agarose gel with TAE buffer, stained with ethidium bromide (5mg/uL), and visualized in a transilluminator.

### Analysis of the characteristics of the vicuñas

To describe the characteristics of the affected vicuñas, a questionnaire was filled out based on information from the animal captured during the “chaccu”. This included data such as age (Pupling: Vicuña up to one year old; Juvenile: Vicuña between 1-3 years of age; Adult: Vicuñas with more than 3 years of age), and sex. The severity of skin lesions was noted as follow: (i) Mild: presence of small skin lesions, (ii) Moderate: with widespread skin lesions in less than 80% of the animal, (iii) Severe: with widespread skin lesions in more than 80% of the animal, with marked alopecia and hyperkeratosis. Furthermore, body conditions were scored using a technique previously used for alpacas: (i) Poor: perceptible vertebrae to the touch of the transverse and spinous processes are pointed, severely concave between the vertebral column and the ribs, (ii) Regular: on palpation, the processes are protruding but smooth, a moderate layer of fat can be felt on the pelvis, slightly concave between the ribs and the spine, (iii) Good: the ribs feel rounded to the touch, the spinous and transverse processes are rounded and neither concave nor convex between the spine and the ribs (Gandarillas, 2016; Beck, 2020). Vicuña´s handling was classified as free-ranging (wildlife without veterinary assistance) and semi-captivity (wildlife with veterinary support during chaccus).

### Statistical analysis

The information on the characteristics of the vicuñas included in the study was analyzed using descriptive statistics, including absolute and relative frequencies, applying confidence intervals at 95%. Association between type of handling with sex, age, injury severity,and body condition in mangy vicuñas were evaluated using the chi-square test (significance level P < 0.05). Statistical analysis was performed using STATA 17.0 for Windows (StataCorp LP, College Station, TX; USA).

## Results

**Figure 3:**
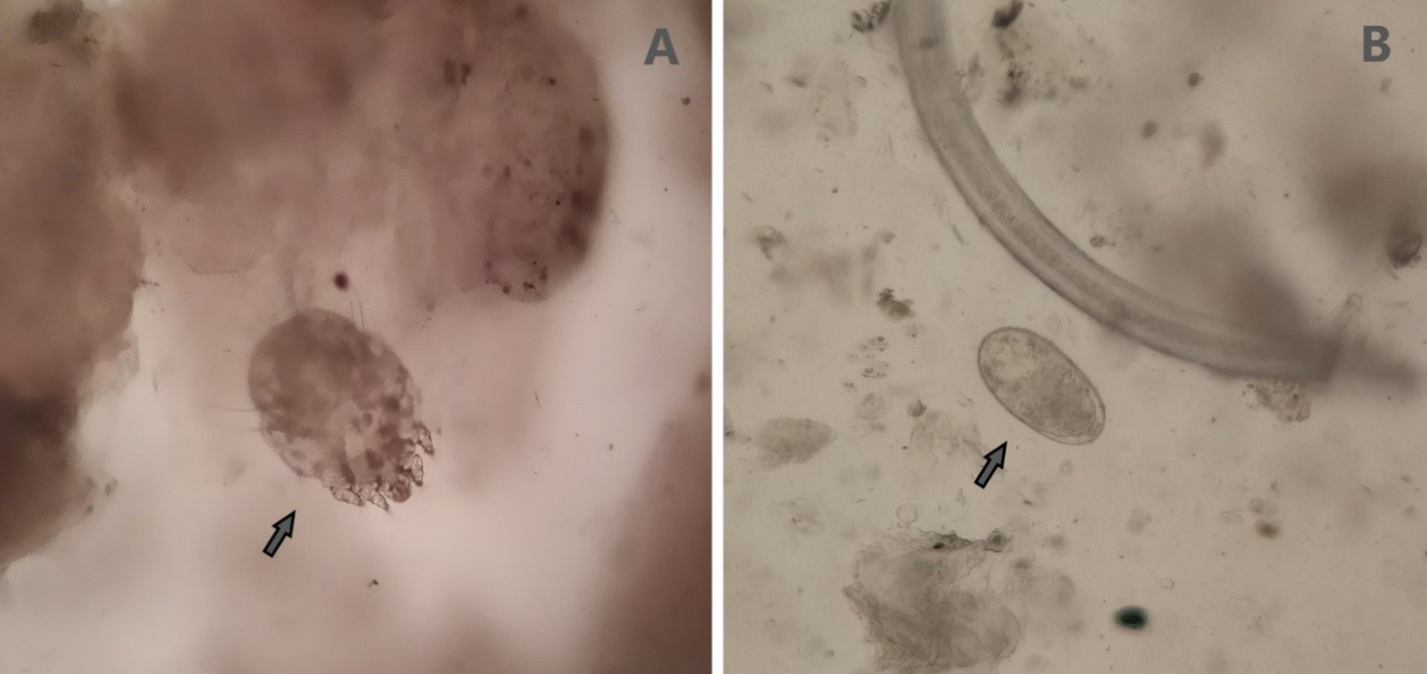
Presence of *Sarcoptes* in vicuña skin scraping samples identified by 40X microscopy. A: adult stage of *Sarcoptes*; B: Egg of *Sarcoptes*.

Out of the 3,274 vicuñas captured, 4.9% (95% CI: 4.1 - 5.6%) showed mangy lesions with the presence of *S. scabiei* (Figure 3). No other genera of mange-producing mites in vicuñas were identified by microscopy analysis. The highest prevalence was found in the highland communities of Lucana (21.8%), Aucara (11.1%), and Cabana (10.8%), all belonging to the department of Ayacucho (Table 1).

**Table 1:** Prevalence of scabies in vicuñas in Andean highland communities in Peru.

**Department**	**Handling type**	**Height**	**Andean highland community**	**Positive**			**Negative**		
				**n**	**(%)**	**CI 95%** ^*^	**n**	**(%)**	**CI 95%**
Junin	Free-ranging and Semi-captivity	4100	Villa de Junin	8	1.3	0.5 - 2.5	601	98.7	97.4 - 99.4
	Free-ranging and Semi-captivity	4100	Ondores	1	0.5	0.1 - 2.6	207	99.5	97.4 - 99.9
Huancavelica	Free-ranging and Semi-captivity	3527	Carhuapata	4	1.7	0.5 - 4.2	233	98.3	95.7 - 99.5
Lima	Semi-captivity	2600	San Pedro de Pilas	0	0.0	0 - 2.1	264	100.0	98.1 - 100
Ayacucho	Free-ranging	3455	Andamarca	14	6.9	3.8 - 11.3	188	93.1	88.6 - 96.2
	Free-ranging and Semi-captivity	3227	Aucara	51	11.1	8.4 - 14.3	407	88.9	85.6 - 91.6
	Free-ranging and Semi-captivity	3310	Cabana	17	10.8	6.4 - 16.6	141	89.2	83.3 - 93.6
	Semi-captivity	3450	Huanca sancos	16	8.3	4.8 - 13.2	176	91.7	86.8 - 95.2
	Free-ranging	3366	Lucanas	12	21.8	11.8 - 35	43	78.2	64.9 - 88.1
	Free-ranging	3570	San Cristóbal	16	3.5	2 - 5.6	441	96.5	94.3 - 97.9
	Free-ranging and Semi-captivity	2627	Uruiza	13	6.8	3.7 - 11.4	177	93.2	88.5 - 96.3
Apurímac	Free-ranging	3553	Huarccoy	2	2.6	0.3- 9.1	74	97.4	90.8 - 99.7
Cusco	Free-ranging	4700	Phinaya	5	3	0.9 - 6-8	163	97.0	93.2 - 99.1
Total				159	4.9	4.1 - 5.6	3115	95.1	94.4 - 95.8

* 95% Confidence Interval.

All vicuña wallows (9/9) were positive for the presence of *Sarcoptes* mites. No other genera of mites were identified based on the microscopy analysis of the soil samples of vicuña wallows. The average parasitic load in the wallows was 0.74 mites per 100 g of soil sample (Table 2).

**Table 2:** Presence of *Sarcoptes* in soils of vicuña wallows with scabies in Andean highland communities in Peru.

**Peasant community**	**Soil type**	**n**	**n (+)**	**Load on 100 g.**
Huarccoy	Clay-Stone	3	2	0.67 *Sarcoptes*
Andamarca	Clay-Stone	3	1	0.33 *Sarcoptes*
San Cristóbal	Clay-Stone	3	4	1.33 *Sarcoptes*
Huarccoy	Clay-Stone	3	2	0.67 *Sarcoptes*
Andamarca	Clay-Stone	3	4	1.33 *Sarcoptes*
San Cristóbal	Clay-Stone	3	2	0.67 *Sarcoptes*
Huarccoy	Clay-Sandy	3	1	0.33 *Sarcoptes*
Andamarca	Clay-Stone	3	2	0.67 *Sarcoptes*
San Cristóbal	Clay-Stone	3	2	0.67 *Sarcoptes*

n: Number of aliquots analyzed in each soil sample, n (+): Number of *Sarcoptes* found in the soil aliquots.

**Figure 4:**
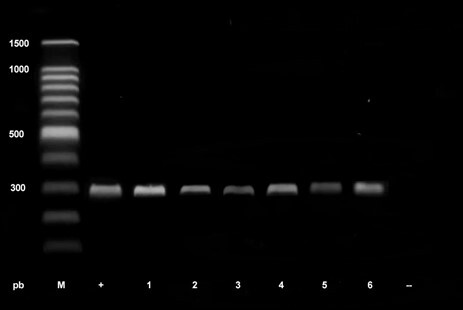
Products amplified by PCR with primers for *Sarcoptes scabiei* in samples of vicuña skin scrapings from Andean highland communities in Peru. An amplicon of 300 base pairs is interpreted as a positive result. M: Marker weight, +: positive control, -Negative control, 1: Andamarca sample, 2: Aucara sample, 3: Cabana sample, 4: San Cristóbal sample, 5: Villa de Junín sample, 6: Sample from Lucana.

Molecular analysis by PCR confirmed that all examined samples from mangy individuals (100/100) were positive for *S. scabiei* by amplification of a specific band of approximately 300 bp in the electrophoresis (Figure 4).

**Figure 5:**
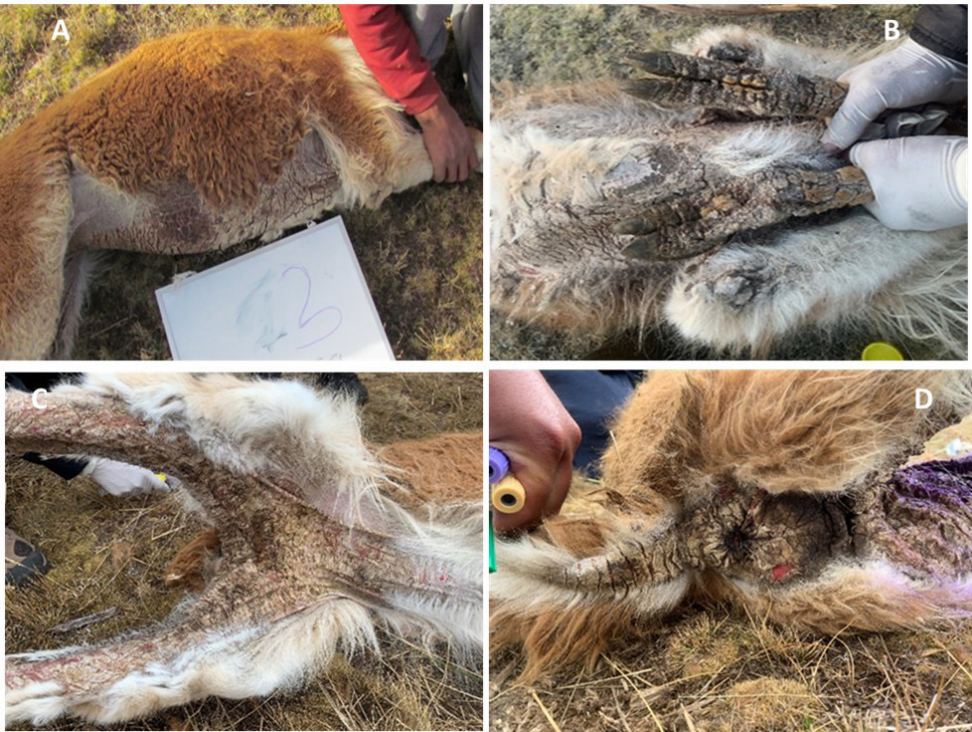
Vicuñas with skin lesions compatible with scabies in distinct locations of the body: (A) Abdomen, (B) Legs, (C) Groin, (D) Anus.

Amongst mangy vicuñas, a higher proportion was represented by females (66%), adults (74.2%), individuals showing mild lesions (42.8%), and individuals with regular body condition (64.2%). The type of handling was not associated with age, sex, injury severity, and body condition (p ≥ 0.05) (Table 3; Figure 5).

**Table 3:** Type of handling and characteristic of mangy *Vicugna vicugna* in Andean highland communities in Peru.

**Variable**	**Category**	**Total**	**Free-ranging**		**Semi-captivity**		*P - value
			**n**	**%**	**n**	**%**	
Sex	Female	105	45	68.2	60	64.5	0.631
	Male	54	21	31.8	33	35.5	
Age	Breeding	11	7	10.6	4	4.3	0.218
	Youth	30	10	15.2	20	21.5	
	Adult	118	49	74.2	69	74.2	
Injury severity	Mild	68	31	47.0	37	39.8	0.392
	Moderate	59	25	37.9	34	36.6	
	Severe	32	10	15.2	22	23.7	
Body condition	Bad	12	4	6.1	8	8.6	0.288
	Regular	102	39	59.1	63	67.7	
	Well	45	23	34.8	22	23.7	

* P-value calculated with the Chi Square test.

## Discussion

This study was designed to identify the presence of mange-producing mites, determine their prevalence, and link these to the characteristics of mangy vicuñas. Results were based on a robust sample size, larger than those of any previous survey. Importantly, the study involved representative samples from the departments with the largest populations of Peruvian vicuñas. In addition, we assessed the impact of management practices (free-ranging or semi-captivity) on mange in vicuñas, a topic that has not been explored by other researchers.

Our findings show an overall prevalence of 4.9% of mange in free-ranging vicuñas among the thirteen highland communities evaluated. This prevalence was higher than those obtained in other studies carried out in Peru. For example, Angulo-Tisoc et al. (2021) analyzed samples of vicuñas in captivity from the province of Canchis, Cuzco region, and reported a prevalence of 0.2% (6/2049). Yet our overall prevalence was much lower than records in other populations of wild vicuñas. For example, Unzueta (2018) found a prevalence of 9.4% (69/733) in the provinces of Andahuaylas and Aymaraes, in Apurímac Region. Castillo Doloriert et al. (2019) reported a prevalence of 37% in San Antonio de Tanta, Lima region. A study carried out on vicuñas from the 'Pampas Galeras' National Reserve found a frequency of mange of 12.0% (24/200) (Gómez-Puerta et al., 2013). In Bolivia, prevalence values ranged between 5.6 and 46.2% (Ruiz, 2016; Beltrán-Saavedra et al., 2011), and in Argentina between, 0.8 and 9.0% (Acebes et al., 2022; Arzamendia et al., 2012). Ferreyra et al. (2022) showed the devastating effects of scabies on wild South American camelids (vicuña and guanaco), observing a reduction in the number of vicuñas close to 70% and detecting the presence of *S. scabiei* in 94% of dead animals and in a considerable part of the few surviving animals.

The low prevalence in this study is possibly explained in that we analyzed wild animals that once a year, during the “chaccu”, are under veterinary care and receive ivermectin, among other treatments, unlike unmanaged wild vicuñas (Angulo-Tisoc et al., 2021). Likewise, the use of antiparasitic drugs, without veterinary supervision, increases the risk that mite resistance may develop (Astorga et al., 2018; Gómez-Puerta et al., 2013; Hunter et al., 2004; Lusat et al., 2009; Pollock et al., 2017).

When it comes to wallows, our study revealed the presence of *S. scabiei* in all tested samples and suggests that they are hotspots for the direct but also indirect transmission of the mites. In this regard, the transmission of mange in vicuñas also could depend on the social behaviors, where vicuñas families dust the fiber in wallows to be protected from cold and prevent felting (Devenish-Nelson et al., 2014). Additionally, there is limited attention to handling procedures and hygienic conditions during the ‘chaccu’ capturing process (Acebes et al., 2022; Arzamendia et al., 2012; Ferreyra et al., 2022; Gómez-Puerta et al., 2013). More studies should be carried out on the epidemiological significance of these hotspots and wallows should be prudentially considered within sanitary control programs.

When it comes to molecular identification, our study confirms that, in the study area, *S. scabiei* is the sole causal agent of mange in vicuñas and that PCR is a reliable and sensitive diagnostic tool (Bae et al., 2020). Our results agree with Gomez-Puerta et al., (2022), who identified *S. scabiei* in mangy vicuñas by analyzing ITS2 and cox1 sequences.

Many studies evaluated the prevalence of scabies and their association with characteristics of the affected vicuñas (sex, age, body condition, severity of lesions, among others) showing inconclusive results. Regarding the characteristics of vicuñas with mange, the frequency of animals affected was higher in females (65.4%) compared to males (34.6%). This result is similar to those Angulo-Tisoc et al. (2021) with 40.7% prevalence in males and 59.2% in females, but different from those reported by Unzueta (2018), who recorded a higher frequency in males. Gómez-Puerta et al. (2022) do not report differences, by sex or age, between vicuñas affected with *S. scabiei* in Cuzco. Although there is no consensus on susceptibility according to sex (Acebes et al., 2022), in Argentina, female vicuñas show a higher level of cortisol during the capture (Marcoppido et al., 2018) and this can affect the immune response, giving greater susceptibility to scabies. Furthermore, females, unlike males, spend more time grouped together, in close contact with their offspring, and near the areas of wallowing, where we have identified the presence of mites (Siguas et al., 2022).

Respect to injuries, we report that minor injuries (74.9%) were more frequent than serious ones. The high frequency of lesions in the affected areas is consistent with what was described by Siguas et al. (2022), which indicates that *S. scabiei* is found primarily in areas devoid of fiber and gradually extends to the medial part of the thighs, legs, and legs interdigital spaces, where 85.7% (12/14) of vicuñas had lesions in the abdomen, armpits, groin, and thorax. While severe infestations can lead to secondary infections, starvation, dehydration, and death, lightly infested individuals may suffer only short-term negative effects (Bornstein & de Verdier, 2010; Twomey et al., 2009). Mange infections have an elevated metabolic cost for severely affected animals that exhibit poor body conditions (Beeler & Applegate, 2021). This helps to explain the relatively low percentage (20.8%) of animals suffering severe injury and the elevated (92.5%) percentage of vicuñas showing a regular body condition in our study.

About the age, our results differ from data reported by Bujaico (2018) which indicates a higher prevalence in juveniles (14.56%) compared to adults (7.24%). Yet the results of our study coincide with those of Unzueta (2018) and Angulo-Tisoc et al. (2021) which found that the highest frequency of mange cases occurred in adult animals. However, our analysis solely focuses on animals that tested positive for mange, whereas the authors mentioned above identified both positive and negative samples for *S. scabiei*. This limitation restricts the scope of conclusions drawn from our study.

## Conclusions

*S. scabiei* was identified as the causal agent of mange in vicuñas from thirteen highland communities from Peru. Mites of the genus *Sarcoptes* were also present in the vicuña wallows, suggesting a likely role of these sites in the indirect transmission of this ectoparasite. The prevalence of sarcoptic mange was low, and many individuals showed mild lesions and a limited effect on body condition.
